# Influence of previous delivery mode on perineal trauma risk

**DOI:** 10.1002/ijgo.14218

**Published:** 2022-05-22

**Authors:** Elizabeth P. C. Thorne, Constantin M. Durnea, Philip M. Sedgwick, Stergios K. Doumouchtsis

**Affiliations:** ^1^ Institute of Medical and Biomedical Education St George’s, University of London London UK; ^2^ Luton and Dunstable University Hospital, NHS Foundation Trust Luton UK; ^3^ Department of Obstetrics and Gynaecology Epsom and St Helier University Hospitals NHS Trust Epsom UK; ^4^ Laboratory of Experimental Surgery and Surgical Research N.S. Christeas National and Kapodistrian University of Athens, Medical School Athens Greece; ^5^ American University of the Caribbean School of Medicine Pembroke Pines Florida USA; ^6^ School of Medicine, Ross University Miramar Florida USA

**Keywords:** cesarean section, obstetric anal sphincter injuries, perineal tear, previous mode of delivery, risk factors, vaginal birth after cesarean

## Abstract

**Objective:**

To evaluate the impact of a previous pregnancy and delivery on perineal trauma rates in the subsequent vaginal birth.

**Methods:**

Retrospective cohort study. The perineal outcomes of secundiparous women with history of previous (first) delivery in one of three categories: failed operative vaginal delivery (FOVD) and second‐stage emergency cesarean section (EmCS); elective cesarean section (ElCS), and vaginal delivery (VD) with intact perineum, were compared with a control primiparous group.

**Results:**

The percentage obstetric anal sphincter injuries (OASIS)at first vaginal delivery was 17.3% (*n* = 9) after previous FOVD+EmCS, 12.9% (*n* = 18) after previous ElCS, and 0.6% (*n* = 9) after previous VD maintaining an intact perineum, compared with 6% (*n* = 1193) in the control primiparous group of women. Multivariate regression analysis demonstrated that previous FOVD+EmCS and ElCS were associated with a statistically significant increased risk of OASIS of 180% and 110% when compared with control (odds ratio [OR] 2.80; 95% confidence interval [CI] 1.35–5.78 and OR 2.10; 95% CI 1.27–3.48, respectively). Previous VD with intact perineum was associated with a statistically significantly reduced risk of OASIS (OR 0.09; 95% CI 0.04–0.17).

**Conclusions:**

Previous FOVD+EmCS and ElCS were associated with increased risk of OASIS in subsequent vaginal delivery compared with control, whereas previous VD with intact perineum was associated with decreased risk.

## Synopsis

Previous emergency cesarean following failed instrumental delivery or elective cesarean is associated with increased risk of obstetric anal sphincter injuries in subsequent vaginal delivery.

## 1 INTRODUCTION

Third‐ and fourth‐degree perineal trauma, alternatively called obstetric anal sphincter injuries (OASIS), is a complication of vaginal delivery. Over the past two decades there has been an increase in the incidence of OASIS, with some series demonstrating a three‐fold increase from 1.8% to 5.9% between 2000 and 2012.^1^

Third‐ and fourth‐degree perineal trauma is known to be associated with potentially major physical and psychological morbidity and long‐term effects.^2^, ^3^, ^4^ Identifying and modifying risk factors is an important prevention strategy. Among recognized risk factors for OASIS are instrumental delivery, ethnicity, use of regional analgesia, gestational age, birth weight, and sex of baby.^5^, ^6^, ^7^ However, the effects of previous pregnancy, labor stages, and delivery methods on the risk of OASIS in subsequent pregnancies are not well known. A previous pregnancy (without the involvement of labor and vaginal delivery), a previous pregnancy and labor (but no vaginal delivery), and a previous vaginal delivery with intact perineum are common obstetric histories in multiparous women. We hypothesized that such histories may be associated with altered incidence of OASIS. This is because a previous pregnancy with the associated hormonal changes may have an effect on the biomechanical properties of the pelvic floor tissues, possibly influencing the risks of perineal trauma in a subsequent pregnancy.

There is a paucity of evidence regarding the impact of the previous mode of delivery on severe perineal trauma (OASIS) in subsequent pregnancy [. Recent studies have evaluated the risk of OASIS in women with previous perineal trauma, in particular those delivering by vaginal birth after cesarean (VBAC).^8^, ^9^, ^10^, ^11^, ^12^ However, the risk of OASIS is unclear, as their results are contradictory. D’Souza et al.^9^ reported a 1.4‐fold increase of OASIS after VBAC compared with primiparous women, and a protective role of mediolateral episiotomy as well as higher incidence if the previous cesarean section (CS) was urgent.^9^ Rusavy et al.^12^ found no difference in the risk of sustaining OASIS between primiparous women and those with a previous CS.

The primary objectives of the present study were to evaluate the impact of a previous pregnancy and different modes of delivery with and without labor on the rates of perineal trauma in the subsequent vaginal birth. In particular:

1. Does a previous pregnancy but no labor or vaginal birth have any impact on the rates of OASIS in the subsequent pregnancy and vaginal birth?
2. Does a previous pregnancy and labor but no vaginal birth have any impact on the rates of OASIS in the subsequent pregnancy and vaginal birth?
3. Does a previous pregnancy, labor, and vaginal birth but no perineal trauma have any impact on the rates of OASIS in the subsequent pregnancy and vaginal birth?

In order to address the primary objectives, we considered the following groups: (1) a previous pregnancy with labor including active second stage (pushing) but no vaginal delivery on OASIS rates in subsequent vaginal delivery. (2) A previous pregnancy but no labor (elective CS) on OASIS rates in subsequent vaginal delivery; and (3) a previous vaginal birth with intact perineum and no diagnosed perineal injury on OASIS rates in subsequent vaginal delivery.For the purposes of the present study, we evaluated the incidence of OASIS, as denoted by third‐ and fourth‐degree perineal tears.

## 2 MATERIALS AND METHODS

The study was conducted in a London tertiary maternity unit. Ethical approval was not required (https://www.hra.nhs.uk/covid‐19‐research/guidance‐using‐patient‐data/#research) because this retrospective cohort study was based on analysis of data collected during standard clinical practice during the study period. All births between the years 1999 and 2015 were reviewed. Anonymized data were acquired from the electronic maternity database and included demographic information, obstetric history, data on first and subsequent pregnancies, parity, gestation, labor and delivery information, mode of delivery, occurrence and type of perineal trauma, use of spinal/epidural analgesia, sex of baby, and birth weight. Women were included if they had been primiparous for their first birth and also had their second child at the tertiary maternity center, while fulfilling the inclusion and exclusion criteria outlined below.

Inclusion criteria were: para two or less, to fit the study design; maternal age between 18 and 40 years; maternal body mass index (BMI; calculated as weight in kilograms divided by the square of height in meters) between 17.5 and 40.0; birth weight less than 5000 g; and gestational age at birth 37–42 weeks. The exclusion criteria were: previous perineal injury; intrauterine fetal death; multiple pregnancies; and emergency CS (EmCS) when unable to confirm that they were performed because of failed operative vaginal deliveries (FOVD).

These inclusion and exclusion criteria were chosen to minimize the potential effect of confounders, and to permit an accurate evaluation of how previous delivery mode can impact perineal trauma in a subsequent delivery. In particular, women under the age of 18 years will not have reached full maturation while a maternal age over 40 years is associated with increased perineal trauma.^13^ Pre‐pregnancy BMI is associated with birth weight of the fetus,^14^, ^15^ and it has been reported that increased maternal BMI is associated with gestational diabetes and a macrosomic fetus.^16^ Women who delivered a fetus that had died in utero were excluded because this is known to be associated with a decreased risk of OASIS,^11^ and may therefore confound the findings.

To investigate the effect of a first pregnancy, labor, and CS or vaginal delivery on the perineum (which had not previously sustained any trauma) the modes of delivery resulting in an intact perineum were identified. These included:

- Group 1: Failed operative vaginal deliveries completed by emergency CS at second stage of labor (FOVD+EmCS),
- Group 2: Elective CS (ElCS),
- Group 3: Spontaneous vaginal delivery (VD) with an intact perineum.

A control group was included to facilitate the comparison with women who had not previously had physiologic pregnancy changes.

- Group 4 (control group): Primiparous women who have a vaginal delivery.

These groups allowed for the analysis of the risk of OASIS in women who had had a vaginal delivery but with differing histories of the extent to which their bodies had previously experienced the physiologic changes of pregnancy and labor. This includes a previous birth without perineal trauma or by abdominal delivery with or without labor.

Logistic regression analysis was undertaken to obtain the unadjusted odds ratios (OR) for risk of OASIS in second pregnancy for groups 1–3 based on mode of delivery in first pregnancy. Adjusted OR were subsequently obtained for the risk of OASIS in the second pregnancy for groups based on mode of delivery in the first pregnancy, having adjusted for the maternal characteristics (age, ethnicity, and BMI), intrapartum risk factors (gestational age at delivery, epidural use, and mode of delivery), plus neonatal measurements (birth weight and sex). Maternal age, maternal BMI, plus birth weight were modeled as categorical variables, with maternal age based on categories of 5 years, BMI on categories of 5 units (kg/m^2^), and birth weight on categories of 500 g. Gestation was modeled as a continuous variable based on number of weeks. The critical level of significance was set at 0.05. No adjustment was made for multiple hypothesis testing. All analyses were performed using SPSS, version 21 (IBM).^17^ For each variable, the number of non‐missing values was used.

## 3 RESULTS

Between 1999 and 2015 there was a total of 74 184 births. Of these, 33 033 births were considered for inclusion in the study. These included cases with a previous FOVD+EmCS in first pregnancy, ElCS in the first pregnancy, previous vaginal birth in first pregnancy maintaining an intact perineum, plus primiparous women who delivered their first child vaginally.

After application of the inclusion and exclusion criteria 21 535 women were included in the study as follows:

Group 1 (FOVD+EmCS): Women with a previous FOVD+EmCS in first pregnancy: 52 (84%) out of 62 met the inclusion criteria.

Group 2 (EICS): Women with ElCS in the first pregnancy: 139 (86%) out of 162 met the inclusion criteria.

Group 3 (SpontaneousVD): Women with a previous vaginal birth in first pregnancy maintaining an intact perineum: 1554 (29%) out of 5425 met the inclusion criteria.

Group 4 (Control): Primiparous women who delivered their first child vaginally: 19790 (73%) out of 27 384 births met the inclusion criteria.

Maternal demographics for the four groups are shown in Table 1. The distribution of BMI was similar between the groups, with mean BMI ranging from 23.9 (control group) to 24.6 (spontaneous VD). The four groups had a diverse ethnic composition. However, the majority of pregnancies were to mothers of white ethnicity in each group with the percentage ranging from 56.7% (*n* = 881) (spontaneous VD) to 71.2% (*n* = 37) (FOVD+EmCS) There was variation between the groups in maternal age; the mean maternal age for both the spontaneous VD group plus the control group was 29.4 years, compared with 33.0 years for the ElCS group.

**TABLE 1:** Maternal demographics^a^

Group number	Group 1	Group 2	Group 3	Group 4
	Previous FOVD+EmCS^b^ (*n* = 52)	Previous ElCS^c^ (*n* = 139)	Previous VD (maintaining an intact perineum) (*n* = 1554)	Primiparous (control group) (*n* = 19 790)
Age, years	31.9 ± 5.1	33.0 ± 4.5	29.4 ± 5.5	29.4 ± 5.1
Median	32.7	33.8	29.4	30.2
BMI^d^	(*n* = 46)	(*n* = 126)	(*n* = 1405)	(*n* = 18 023)
	24.5 ± 3.7	24.0 ± 3.6	24.6 ± 4.3	23.9 ± 5.4
Median	23.9	23.4	23.8	23.1
Ethnicity				
White	37 (71.21%)	93 (66.9%)	881 (56.7%)	11 723 (59.2%)
Black	3 (5.8%)	7 (5.0%)	266 (17.1%)	2030 (10.3%)
Asian	9 (17.3%)	30 (21.6%)	225 (14.5%)	3581 (18.1%)
Other	3 (5.8%)	9 (6.4%)	182 (11.7%)	2456 (12.4%)

Abbreviations: BMI, body mass index (calculated as weight in kilograms divided by the square of height in meters); ElCS, elective cesarean section; EmCS, emergency cesarean section; FOVD, failed operative vaginal delivery; VD, vaginal delivery.

^a^ Values are presented as mean ± standard deviation; median, or number (percentage, representing the percentage of pregnancies within group).

^b^ Failed operative vaginal delivery and emergency cesarean section.

^c^ Elective cesarean section.

^d^ Incomplete data available for BMI.

Neonatal characteristics for each of the four groups are shown in Table 2. The primiparous control group had the lowest mean birth weight (3334.7 g) compared with the FOVD+EmCS group that had the largest mean birth weight (3539.8 g). The percentage of male neonates was higher than that of females for all birth groups, ranging from 50.4% (*n* = 70) (previous ElCS) to 55.8% (*n* = 29) (previous FOVD+EmCS group).

**TABLE 2:** Neonatal characteristics^a^

Group number	Group 1	Group 2	Group 3	Group 4
	Previous FOVD+EmCS^b^ (*n* = 52)	Previous EICS^c^ (*n* = 139)	Previous VD (maintaining an intact perineum) (*n* = 1554)	Primiparous (control group) (*n* = 19 790)
Sex				
Male	29 (55.8%)	70 (50.4%)	823 (53.0%)	9994 (50.5%)
Female	23 (44.2%)	69 (49.6%)	730 (47.0%)	9796 (49.5%)
Birth weight, g	3539.8 ± 473.6	3414.1 ± 481.1	3383.7 ± 454.8	3334.7 ± 437.3
Median	3585	3400	3400	3320

Abbreviations: ElCS, elective cesarean section; EmCS, emergency cesarean section; FOVD, failed operative vaginal delivery; VD, vaginal delivery.

^a^ Values are presented as mean ± standard deviation; median, or number (percentage, representing the percentage within the group).

^b^ Failed operative vaginal delivery and emergency cesarean section.

^c^ Elective cesarean section.

Pregnancy and birthing outcomes for each of the four groups are shown in Table 3. The group of women who had a previous VD with no OASIS were more likely to deliver via spontaneous VD, with only 3.9% (*n* = 56) requiring operative intervention.

**TABLE 3:** Pregnancy and birth outcomes^a^

Group number	Group 1	Group 2	Group 3	Group 4
	Previous FOVD+EmCS^b^ (*n* = 52)	Previous ElCS^c^ (*n* = 139)	Previous VD (maintaining an intact perineum) (*n* = 1554)	Primiparous (control group) (*n* = 19 790)
Gestation, week	40.3 ± 1.0	40.1 ± 1.2	39.9 ± 1.1	40.1 ± 1.1
	40 (40–41)	40 (39–41)	40 (39–41)	40 (39–41)
Epidural analgesia	17 (32.7%)	73 (52.5%)	190 (12.2%)	8496 (42.9%)
Birth type				
Spontaneous VD	28 (53.8%)	89 (64.1%)	1498 (96.4%)	13 322 (67.4%)
Ventouse	13 (25.0%)	31 (22.3%)	48 (3.1%)	4365 (22.1%)
Failed ventouse to forceps	3 (5.8%)	6 (4.3%)	2 (0.1%)	698 (3.5%)
Forceps	8 (15.5%)	13 (9.4%)	6 (0.7%)	1405 (7.1%)
Perineal trauma				
Intact perineum	2 (3.8%)	15 (10.8%)	1140 (73.4%)	3504 (17.7%)
First‐degree	5 (7.7%)	13 (9.4%)	167 (10.7%)	1733 (8.8%)
Second‐degree	27 (32.7%)	49 (35.3%)	197 (12.7%)	7094 (35.8%)
Third‐degree	9 (17.3%)	17 (12.2%)	9 (0.6%)	1167 (5.9%)
Fourth‐degree	0 (0%)	1 (0.7%)	0 (0%)	26 (0.1%)
Episiotomy	20 (38.5%)	44 (31.7%)	41 (2.6%)	6266 (31.7%)
Intact perineum	2 (3.8%)	15 (10.8%)	1140 (73.4%)	3504 (17.7%)

Abbreviations: ElCS, elective cesarean section; EmCS, emergency cesarean section; FOVD, failed operative vaginal delivery; VD, vaginal delivery.

^a^ Values are presented as mean ± standard deviation; median (interquartile range), or as number (percentage, representing the percentage within the group).

^b^ Failed operative vaginal delivery and emergency cesarean section.

^c^ Elective cesarean section.

The effect of previous pregnancy and mode of delivery on risk of OASIS was shown by the percentage OASIS being 17.3% (*n* = 9) at first vaginal delivery after previous FOVD+EmCS, 12.9% (*n* = 18) after previous ElCS, and 0.6% (*n* = 9) after previous VD with intact perineum, compared with 6% (*n* = 1193) in the control primiparous group of women (Table 4).

**TABLE 4:** Unadjusted and adjusted odds ratios for the risk of OASIS in second pregnancy for each of the four groups based on their mode of delivery in first pregnancy^a^ ^,^ ^b^

Previous mode of delivery	Pregnancies with OASIS (*n* = 1229)	Univariate analysis			Multivariate analysis^c^		
		OR	95% CI	*P* value	OR	95% CI	*P* value
Group 1: Previous FOVD+EmCS (*n* = 52)	9 (17.3%)	3.326	(1.59–6.71)	0.001	2.80	(1.35–5.78)	0.006
Group 2: Previous ElCS (*n* = 139)	18 (12.9%)	2.32	(1.41–3.82)	<0.001	2.10	(1.27–3.48)	0.004
Group 3: Intact perineum with previous vaginal delivery (*n* = 1554)	9 (0.6%)	0.09	(0.05–0.17)	<0.001	0.09	(0.05–0.17)	<0.001
Group 4: First pregnancy vaginal delivery (control group) (*n* = 19 790)	1193 (6.0%)	Reference group			Reference group		

Abbreviations: CI, confidence interval; ElCS, elective cesarean section; EmCS, emergency cesarean section; FOVD, failed operative vaginal delivery; OR, odds ratio; VD, vaginal delivery.

^a^ The control primiparous group was the reference group. Odds ratios were adjusted for the effects of maternal age, maternal body mass index (calculated as weight in kilograms divided by the square of height in meters), maternal ethnicity, gestation, birth weight, mode of delivery, epidural use and sex of child.

^b^ Values are presented as number (percentage) unless otherwise stated.

^c^ Adjusted for maternal age, maternal body mass index (calculated as weight in kilograms divided by the square of height in meters), maternal ethnicity, gestation, birth weight, mode of delivery, epidural use and sex of child.

The unadjusted and adjusted OR for the risk of OASIS in the subsequent pregnancy for each group based on their mode of delivery in the first pregnancy are presented in Table 4. The control primiparous group was the reference group. OR were adjusted for the effects of maternal age, maternal BMI, maternal ethnicity, gestation, birth weight, mode of delivery, epidural use, and sex of child. Multivariate regression analysis demonstrated that previous FOVD+EmCS and ElCS were associated with a statistically significant increased risk of OASIS of 180% and 110% when compared with control (OR 2.80; 95% confidence interval [CI] 1.35–5.78 and OR 2.10; 95% CI 1.27–3.48, respectively). Previous VD with intact perineum was associated with a statistically significantly reduced risk of OASIS of 91% (OR 0.09; 95% CI 0.04–0.17). There was little difference in magnitude between the unadjusted and adjusted odds ratios, whereas statistical significance remained following adjustment for confounding. This suggests that there is limited confounding due to maternal characteristics, intrapartum risk factors, plus neonatal measurements.

## 4 DISCUSSION

Women with a history in their first delivery of EmCS after FOVD, or ElCS had a significant increase in the risk of OASIS in their subsequent pregnancy when compared with control (180% and 110%, respectively). Those who had a previous vaginal birth maintaining an intact perineum had a reduced risk (0.6%) of OASIS of 91% when compared with control in a subsequent vaginal birth; this suggests that a previous vaginal delivery with intact perineum is a protective factor from OASIS. These findings may have an impact on antenatal counseling and help to inform women’s choices around the preferred mode of delivery when there is relevant obstetric history with factors increasing or decreasing the background risk of OASIS.

A strength of the present study is the classification of the study population into the four groups, allowing specific evaluation of risk of OASIS according to previous mode of delivery, which adds new information to our understanding of under‐studied risk factors associated with perineal trauma. A further strength is the large number of women studied through hospital records with complete data, along with the control for confounding factors. The effect of a series of well‐documented and known risk factors for OASIS were controlled for in the multivariate analyses.^18^ The diagnosis of OASIS is based on clinical criteria as per standard practice, and underdiagnosis or overdiagnosis is possible. However, when compared with ultrasonographic diagnosis according to other researchers,^19^ the cases of OASIS in the present study were always repaired by surgeons with specific training in these types of repairs or supervised by trained surgeons, thus adding confirmation of clinical diagnosis and surgical expertise in the diagnosis and management of these cases. Additionally, all cases of OASIS are regularly reviewed by the Departmental Risk Management Group. Data and medical records in relation to these cases were cross‐checked and scrutinized.

The main drawback was the inherent limitation of the retrospective observational study design. Data were recorded by a variety of staff members with an inherent possible variation of the accuracy of the measurements recorded. However, data entries of OASIS cases are cross‐checked when inputted into the database, reducing the likelihood of errors. Nevertheless, as a result of the wide time frame of data collection, and the inevitable variation in staff collecting data, the study is open to inaccuracies during data input. A further limitation was unknown numbers of women lost to follow up (numbers of women who subsequently delivered at another hospital). Data were collected over a 16‐year period, and it is likely that these numbers were small. However, the groups of previous FOVD+EmCS and ElCS were small in number, so any reduction in numbers may have a big impact on the precision of estimates with wider confidence intervals. It is worth noting that some data were partially missing, particularly maternal BMI. Lack of data on indication for a previous CS was a further limitation. Misdiagnoses, especially of an intact perineum (when there is an occult injury), or misclassifications of perineal trauma on clinical grounds is an inherent limitation in all studies in this field, particularly those based on retrospective data collection.^20^, ^21^, ^22^

There is limited evidence, to our knowledge, on the risk of OASIS in the index pregnancy associated with the mode of delivery in a previous pregnancy. A small number of studies^23^, ^24^ have reported a previous CS as a significant risk factor for OASIS at subsequent vaginal delivery, in agreement with our findings. In line with our findings are also findings by Rusavy et al.^12^ who reported that first vaginal births after CS have an increased association with both perineal and cervical lacerations. Furthermore, D’Souza et al.^9^ demonstrated increased risk of OASIS at time of VBAC depending on the urgency of the initial CS. However, aside from this, there is sparse evaluation of OASIS risks in women with previous EmCS after FOVD and ElCS separately. The present study demonstrated increased rates of OASIS in subsequent vaginal delivery in women having previous EmCS after FOVD compared with those having previous ElCS. This may result from undiagnosed or underreported cephalopelvic disproportion, which may be recurrent and contribute to an increased risk of OASIS in subsequent vaginal births.^23^ A group of primiparous women were selected to provide a control group with the baseline rates of OASIS.

In conclusion, the present study provides additional information on risk factors for perineal trauma. Women having their first vaginal birth after ElCS or an EmCS for failed operative vaginal delivery have a high risk of sustaining OASIS during the subsequent vaginal delivery.

Further research is required to establish the etiology and physiologic or anatomical mechanisms implicated in these clinical and epidemiologic observations. Also, further research may shed light on the OASIS risks by evaluating larger group sizes and subcategories.

These findings could potentially support antenatal counseling. Due to the physical and psychological implications of severe perineal trauma, it is recommended that all women considering a vaginal birth after EmCS following failed instrumental delivery be counseled on the risk of OASIS. Additionally, the associations of forceps delivery, either as a primary mode of delivery or following a failed attempt for ventouse, with risks of OASIS, should be ideally communicated with the woman. However, in most cases where forceps are selected to assist a vaginal delivery, decisions are often made in clinical situations of emergency when time does not permit detailed patient counseling. Therefore, considerations of the best interests of the mother and baby may indicate choices by the obstetrician that would potentially allow for higher risks of perineal injury.

## AUTHOR CONTRIBUTIONS

EPCT contributed to project development, data collection, statistical analysis, manuscript writing, and critical review. CMD contributed to manuscript writing. PMS contributed to statistical analysis, manuscript writing, and consultation. SKD conceived the idea and contributed to project development, data collection, manuscript writing, consultation, and final approval.

## CONFLICT OF INTEREST

The authors declare that they have no conflicts of interest.

## Data Availability

Research data are not shared.
